# Rebound After Fingolimod and a Single Daclizumab Injection in a Patient Retrospectively Diagnosed With NMO Spectrum Disorder—MRI Apparent Diffusion Coefficient Maps in Differential Diagnosis of Demyelinating CNS Disorders

**DOI:** 10.3389/fneur.2018.00782

**Published:** 2018-09-27

**Authors:** Franca Wagner, Lorenz Grunder, Arsany Hakim, Nicole Kamber, Michael P. Horn, Julia Muellner, Robert Hoepner, Roland Wiest, Imke Metz, Andrew Chan, Anke Salmen

**Affiliations:** ^1^Department of Diagnostic and Interventional Neuroradiology, Inselspital, University Hospital and University of Bern, Bern, Switzerland; ^2^Department of Neurology, Inselspital, University Hospital and University of Bern, Bern, Switzerland; ^3^Institute of Clinical Chemistry, Inselspital, University Hospital and University of Bern, Bern, Switzerland; ^4^Institute of Neuropathology, University Medical Center Göttingen, Göttingen, Germany

**Keywords:** MRI, ADC, histogram analysis, MS, multiple sclerosis, neuromyelitis optica, NMOSD

## Abstract

**Objective:** Differential diagnosis of neuromyelitis optica spectrum disorders (NMOSD) and multiple sclerosis (MS) or mimics can be challenging, especially in patients with atypical presentations and negative serostatus for aquaporin-4 antibodies (AQP4-Ab). This brief research report describes magnetic resonance imaging (MRI) findings focusing on quantitative apparent diffusion coefficient (ADC) histogram analysis as a potential tool to differentiate NMOSD from MS.

**Methods:** Longitudinal MRI data obtained during routine clinical examinations were retrospectively analyzed in a patient with histologically determined cerebral NMOSD, a patient with an acute tumefactive MS lesion, and a patient with ischemic stroke. Histogram analyses of ADC maps were evaluated.

**Results:** A patient diagnosed with MS experienced a severe rebound after fingolimod withdrawal and a single daclizumab injection. Cerebral NMOSD manifestation was confirmed by brain biopsy. However, the patient did not fulfill consensus criteria for NMOSD and was AQP4-Ab negative. Comparison of ADC histogram analyses of this patient with those from a patient with MS and one with ischemic stroke revealed differential ADC characteristics: namely a more pronounced and prolonged ADC leftward shift in inflammatory than in ischemic pathology, even more accentuated in NMOSD versus MS.

**Conclusion:** ADC map histograms and ADC threshold values for different conditions may be useful for differentiation of large inflammatory brain lesions and further studies are merited.

## Introduction

Neuromyelitis optica spectrum disorder (NMOSD) and multiple sclerosis (MS) are inflammatory disorders of the central nervous system (CNS) that can have clinical and radiological overlaps. Differential diagnosis can be challenging, especially in patients with NMOSD who test negative for aquaporin-4 antibodies (AQP4-Ab, up to 20%) (1–3). This is a matter of high clinical relevance as MS drugs may not only lack efficacy in patients with NMOSD but might even be harmful (4–7). Additional objective markers that distinguish AQP4-Ab-negative NMOSD from MS are thus needed.

We report on a patient treated for relapsing–remitting MS (RRMS) who experienced a severe rebound of an inflammatory CNS disorder after cessation of fingolimod and overlapping initiation of daclizumab. In this patient with negative serum AQP4-Ab and anti-myelin-oligodendrocyte-glycoprotein (MOG-)Ab, brain biopsy revealed neuropathological characteristics of NMOSD.

Acute demyelinating CNS lesions are described as exhibiting restricted diffusion (8). Nevertheless, routine magnetic resonance imaging (MRI) including diffusion-weighted sequences cannot differentiate between acute inflammatory and ischemic entities. Therefore, textural analysis methods in MRI are receiving increased attention. We discuss the potential use of quantitative apparent diffusion coefficient (ADC) histogram analysis on cranial MRI as a diagnostic tool that may aid in differentiation between MS and NMOSD.

## Methods

The local ethics committee (Kantonale Ethikkommission Bern, Switzerland, KEK-BE reg. no. 2016-02035, 2017-01369) gave approval for access to and use of the patient data for retrospective clinical research. All data presented were obtained during routine clinical investigations and retrospectively analyzed in a pseudonymized fashion.

A retrospective case analysis of an NMOSD patient followed the clinical evolution, paraclinical findings, and MRI results. A longitudinal case study using quantitative MRI analysis was performed to compare the NMOSD patient with an RRMS patient during relapse, and a patient with acute ischemic stroke.

The routine clinical MRI protocol for MS included the following non-contrast sequences: diffusion-weighted imaging, 3D T1w MP-RAGE and 3D T2w GRE. After contrast application, PD/T2w, axial T1w SE, and 3D T1w MP-RAGE were acquired. ADC maps were evaluated longitudinally (b = 0, b = 1,000 s/mm^2^, monoexponential ADC calculation) on a pixel-by-pixel basis for the diffusion-restricted area and used for histogram analysis (9). For the analysis of the ADC maps, we used commercially available software for automated and multivendor postprocessing (Olea Sphere®, version number 3.0-SP11). The lesion voxels collected from the contoured regions of interest (ROIs) provided histograms from which dispersion parameters were computed (9). For all measurements, we used the ADCs provided by the imaging system, with a reference value for water of 2.3 × 10^−9^ m^2^/s.

Each MRI examination generated multiple sections with abnormal ROIs. Skewness and kurtosis derived from the histograms reflected the shape of each histogram. Kurtosis is a measure of the peak of the histogram. It is zero if there is a Gaussian distribution, positive if it has a sharp peak, and negative if it has a flat plateau. Skewness is a measurement of the histogram asymmetry. It is zero when the majority of the data are concentrated in the midline, positive if the majority of data are concentrated to the left of the mean, and negative if the majority of data are concentrated to the right of the mean.

## Results

### Clinical findings

Our patient was diagnosed with RRMS in 2002 after a severe first relapse with hemiparesis on the right side. She was treated with interferon-beta from 2003 until 2015. Owing to insufficient clinical response and continuing relapse activity, as well as side effects, her treatment was switched to fingolimod in 2015. Relapse phenotypes were described by the patient as having included optic neuritis (right) and coordination problems with the right hand. During fingolimod treatment, ongoing MRI activity was detected. In 2016, the fingolimod dosage was reduced due to lymphopenia (0.5 mg, 5 times a week). Since the lymphopenia did not resolve, fingolimod was finally stopped in March 2017 (last documented lymphocyte value 0.38 G/l) and daclizumab was started at the end of April 2017 (documented normalization of lymphocyte count to 1.37 G/l). Only 7 days after the first administration of daclizumab, severe progressive neurological deterioration occurred. The patient initially showed quadrant anopsia (lower right quadrant), which progressed rapidly despite early initiation of high-dose steroids. When she was referred to our hospital only 2 days later, the patient showed hemiplegia with neglect and hemianopsia (right), global aphasia, dysphagia and reduced consciousness (EDSS 9.5).

Over the course of 15 days, the five MR brain scans of our patient conducted within this period revealed markedly progressive T2/FLAIR-hyperintense signal alterations in the left hemisphere from the frontoparietal region to the occipital lobe with increasing patchy contrast enhancement in the confluent lesion.

### Laboratory findings

CSF analysis demonstrated 25 cells/μl with lympho-monocytic pleocytosis and presence of CSF-specific oligoclonal bands (OCB). Highly sensitive polymerase chain reaction (PCR) detected neither JC virus DNA nor HSV or VZV DNA. The results of serological screening for infectious agents were negative (hepatitis B/C, HIV, *Borrelia burgdorferi, Treponema pallidum, Mycobacterium tuberculosis*). Differential screening tests for vasculitis and connectivitis were negative. Repeated testing for AQP4-Ab (cell-based assay: IIFT, Euroimmun AG, Lübeck, Germany) was negative. Retrospective testing of the first plasma exchange (PLEX) specimen was negative for AQP4-Ab and MOG-Ab. All tests were performed after admission to our hospital following a previous pulsed steroid treatment at another location.

### Brain biopsy

Brain biopsy was performed due to further deterioration despite two high-dose steroid pulses and seven sessions of plasma exchange, presenting with a seizure of focal onset. The biopsy showed an inflammatory demyelinating lesion (Supplementary Material). The inflammatory infiltrate consisted of numerous macrophages with macrophage-derived foam cells, and a CD8+-dominated T cell infiltrate. The demyelination was patchy. Strikingly, the lesion showed astropathic changes with a reduction of astrocytes and AQP4 loss within the lesion areas. These changes are typical for NMOSD, but not for MS. Also typical for NMOSD, was loss of oligodendrocytes. CD20-positive B-cells were found in perivascular compartments. Complement or immunoglobulin (Ig) deposits around vessels or eosinophils within lesions—changes observed especially in early active NMOSD lesions—were not present (10). The brain biopsy showed no evidence for progressive multifocal leukoencephalopathy or lymphoma.

### Clinical evolution

The patient initially received acyclovir as a co-treatment; and sequentially high-dose pulsed steroids and PLEX. Beginning clinical remission was noted. Follow-up MRI 1 month later showed that the lesion extent had decreased. With the help of extensive rehabilitation therapy, the patient further improved (last documented EDSS 5.0). Long-term treatment with rituximab was initiated 8 weeks after symptom onset (total dosage 1,500 mg) and the patient exhibited clinical and radiological stability at the time of writing (after approximately 6 months of follow-up).

### ADC histogram: differences between entities

**Figure 1:**
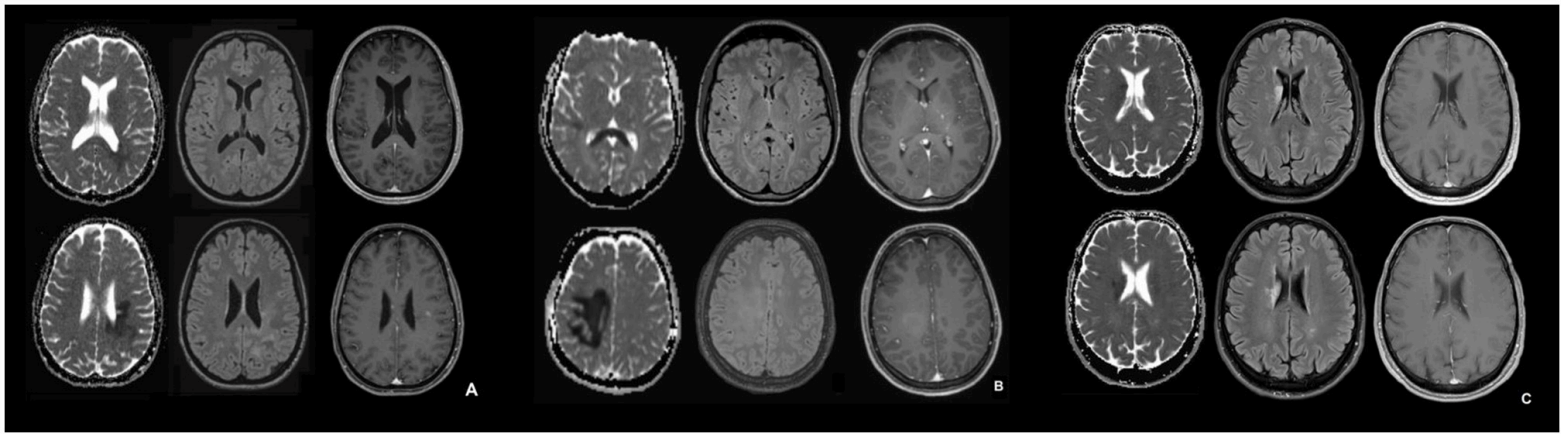
MRI in the acute phase of NMOSD **(A)**, tumefactive MS **(B)**, and ischemic stroke **(C)**. **(A–C)**: Apparent diffusion coefficient (ADC) map (left), transverse FLAIR-attenuated inversion recovery weighted scan (middle), transverse contrast-enhanced T1 weighted scan (right) for the NMOSD patient **(A)**, the patient with tumefactive MS **(B)** and the patient with ischemic stroke **(C)**. In **(B)** the contrast enhancement in basal ganglia on the left corresponds to a developmental venous anomaly.

Head MRI on admission (before biopsy) indicated a large FLAIR hyperintense and diffusion-restricted periventricular white matter lesion located in the parietal, occipital and frontal lobe with faint contrast enhancement in the anterior portion of the lesion (Figure 1A). Spinal MRI showed a non-enhancing T2-hyperintense lesion at the C3–4 level, which had been noted on previous scans, and two focal non-enhancing lesions in the conus medullaris.

To detect potential differences in MRI features between patients with NMOSD, MS, and ischemic stroke, we compared longitudinal MRI data from our NMOSD patient, acquired in the acute phase (day 3 from symptom onset; Figure 1A), with an RRMS patient who had an acute tumefactive MS lesion (Figure 1B) and a patient who had had an acute ischemic stroke (Figure 1C).

In the patient with histopathological features of NMOSD, the ADC maps showed restricted periventricular diffusion in the frontal, parietal and occipital lobe with corresponding signal abnormalities on FLAIR-weighted imaging and mild focal contrast enhancement. Similarly, in the RRMS patient with a tumefactive MS lesion, there was a periventricular ADC restriction in the frontal, parietal, and occipital lobe and in the splenium. This, again, was associated with corresponding signal abnormalities on FLAIR-weighted imaging and diffuse focal contrast enhancement. Quantitative ADC maps for the patient with acute ischemic stroke showed restricted periventricular diffusion in the caudate nucleus and in the frontal lobe, and signal abnormalities on FLAIR-weighted imaging, but no contrast enhancement. The signal characteristics of FLAIR and ADC were similar and thus could not be used to differentiate between the entities.

**Figure 2:**
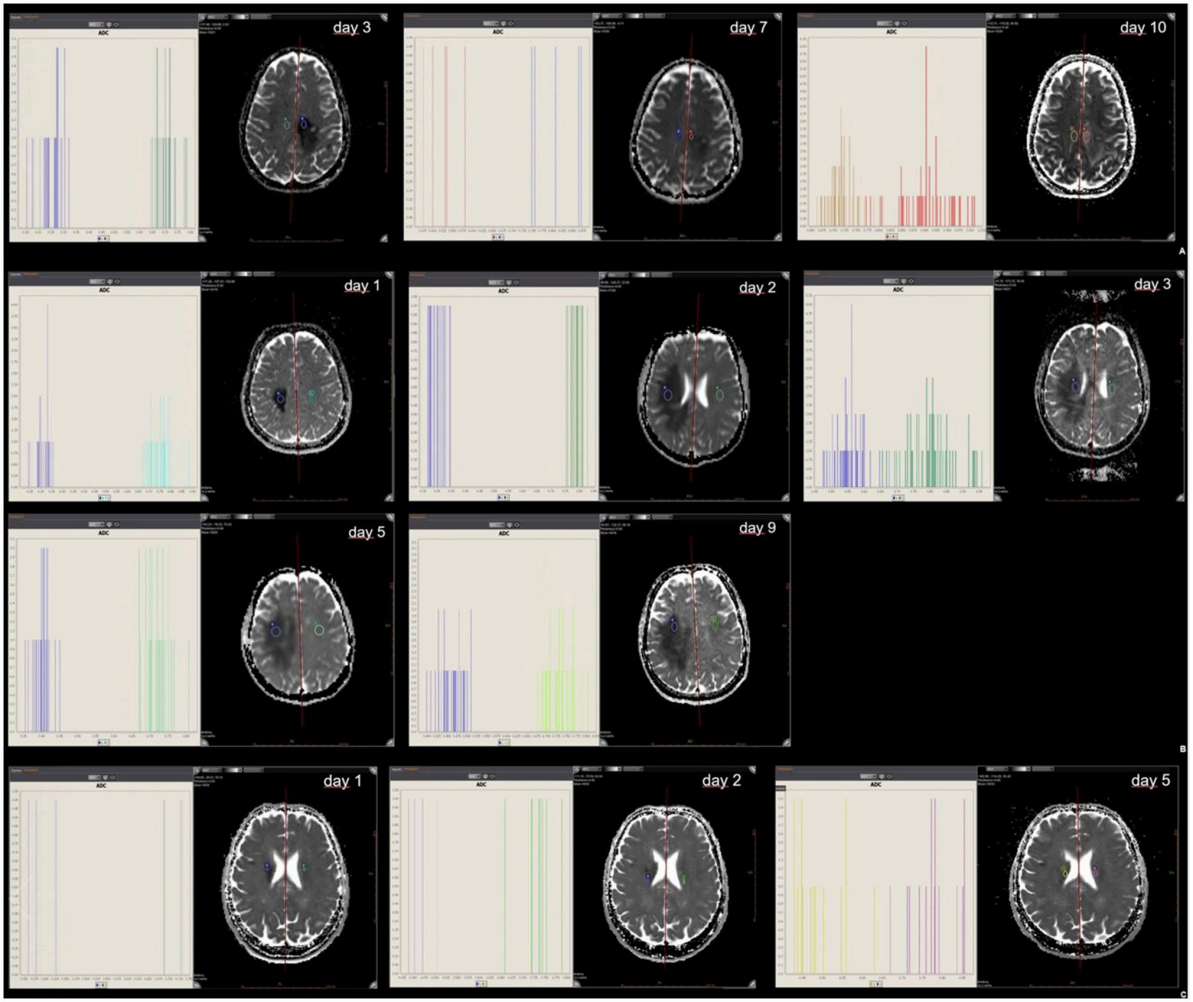
ROI mean ADC signal intensity of NMOSD **(A)**, tumefactive MS **(B)**, and ischemic stroke **(C)**. Mean ADC values of abnormal ROIs in NMOSD (2A), MS (2B) and ischemic stroke (2C) at the time of initial MR and at follow-up; with histogram analysis for comparison.

All three patients underwent several follow-up MRIs with anatomic and diffusion-weighted sequences. The mean ROI-based ADC values, skewness and kurtosis were determined from the MRIs from the patients with brain lesions and ischemic stroke, respectively (see Supplementary Material). ADC values were reduced in NMOSD, MS and ischemic stroke, with a corresponding skewness of the ADC histograms when compared with normal tissue (normal white matter ADC values range from 670 to 800^*^10^−6^mm^2^/s). (11) In this one-to-one, days-after-onset comparison and follow-up, the leftward shift in the NMOSD patient was more distinct and prolonged than those seen in the MS and ischemic stroke patients (Figures 2A–C). Skewness in the patient with ischemic stroke was markedly less pronounced than in those with MS and NMOSD.

## Discussion

We describe a patient with histopathological changes typical of NMOSD with clear astrocytopathy and AQP4 loss. However, this patient did not fulfill the diagnostic consensus criteria for NMOSD, (2) meeting only one clinical core criterion (cerebral manifestation), and showing absence of longitudinal extensive transverse myelitis (LETM) or clear optic neuritis, and AQP4-Ab negativity.

The classical structural pattern of NMOSD is extensive lesions, particularly in the spinal cord (presenting as LETM) and the optic nerve. However, revised diagnostic criteria emphasize other sites of inflammation such as the area postrema, brainstem, and diencephalon, as well as cerebral manifestations (2).

In retrospect, the history of optic neuritis—described by the patient, but not distinctly mentioned in her previous medical records—might have led NMOSD to be considered earlier. However, optic neuritis is a very common symptom in MS too, and only now is MRI able to demonstrate different patterns of optic neuritis in MS versus NMOSD (2). An imaging-based differential diagnosis with older scans would thus have been be highly unlikely.

The patient was seronegative for both AQP4-Ab and MOG-Ab assessed in a cell-based assay. Up to 20% of NMOSD patients are seronegative (3). As immunomodulatory treatments including steroids may influence these results, we cannot exclude false-negative findings (12). This reflects a common clinical dilemma as the diagnostic process often runs in parallel with already administered acute treatments.

In this case, the additional diagnostic findings, especially the CSF results showing lympho-monocytic pleocytosis and presence of CSF-specific OCB, did not help in differentiating NMOSD from MS. For OCB, frequencies up to 30% in NMOSD patients have been reported (3).

Interestingly, in our patient's previous MRI scans, brain pathology before the current attack was not highly suggestive of long-standing MS. Scans over the 15-years disease course demonstrated only a few rather unspecific lesions, except for one larger lesion adjacent to the left ventricle. The overall lesion burden was thus low. The spinal cord had focal, non-LETM lesions. This is usually considered as being MS-specific; however, short transverse myelitis is also a common phenomenon in NMOSD and has been found to delay diagnosis and treatment of NMOSD (13).

NMOSD with cerebral pathology was only differentiated from MS, after 15 years of disease course, by brain biopsy. Even so, not all the described features of NMOSD were found in the biopsy material (10). This again might have been influenced by both the timing of the biopsy and previous administration of acute treatments including steroids and plasma exchange. Eosinophilic infiltration and complement or Ig deposition as markers of early active lesions were thus not detectable. However, the pattern of extensive macrophage infiltration, loss of AQP4, astrocytes and oligodendrocytes, together with perivascular B-cell infiltration was highly suggestive.

The lack of typical LETM or clear optic neuritis and negative AQP4-Ab together with the suboptimal response to treatment with interferon-beta and fingolimod may never have led to consideration of NMOSD, as this is also seen in MS. However, after cessation of fingolimod, severe disease exacerbation was noted. This rebound phenomenon has recently been described in MS patients, (14–16) but also as a potentially severe phenomenon in patients with NMOSD (6). In our patient, rebound occurred sooner than reported in the literature. Early re-initiation of therapy, within 1 month, with a highly active compound, daclizumab, could not prevent fingolimod-associated rebound. As there is no information on IL-2 receptor blockade in NMOSD patients, it is not known whether daclizumab might have propagated the rebound. Given the short latency between the first and only injection of daclizumab and symptom onset, causality seemed unlikely. However, in light of the recent withdrawal from the market of daclizumab following eight cases of serious inflammatory brain disorders, (17) this possibility might need to be reconsidered once further information on these cases becomes available.

Anatomical MRI findings of brain lesions can be typical of both NMOSD and MS (18). In particular, for intracranial NMOSD manifestation, cases with occipital lesions similar to our patient have been reported (19, 20). Involvement of the splenium in an “arch bridge pattern” as seen in the MS patient is actually more prevalent in NMOSD patients (18). Lesion enhancement in seronegative NMOSD patients is more prevalent than in seropositive ones, complicating both radiological and clinical differential diagnosis of large inflammatory brain lesions (21, 22). While asymptomatic or post-acute NMOSD brain lesions typically show high intensity on both DWI and ADC, there is a lack of literature about DWI and ADC imaging characteristics of symptomatic NMOSD brain lesions (23).

We compared the MRI characteristics of our patient to MRIs from patients with MS and ischemic stroke. NMOSD lesions display a loss of astrocytes (20, 24) as was evident in the biopsy material from our patient, which is atypical for MS. However, in anatomical MRI, this is not well represented. Quantitative and longitudinal MR analysis may further aid in this respect (9, 25).

Skewness and kurtosis are relatively simple parameters that describe texture heterogeneity. Computer-aided texture analysis shows promise as a method for lesion characterization. It assesses and quantifies lesion characteristics using pixel values and/or their distribution within target lesions, providing a more detailed and reproducible quantitative assessment than is possible from visual analysis by human observers.

In our case comparison, a difference in ADC map histograms was detected. Skewness in ischemic stroke is markedly less pronounced than in MS and NMOSD. The texture parameter skewness may help to distinguish between these diseases. The difference can be summarized as a more pronounced and prolonged skewness in inflammatory brain pathology than in stroke and seems to be even more pronounced and prolonged in NMOSD than in MS. It is important to bear in mind that these are only observations in single patients and confirmation from larger studies is necessary.

However, in several tumor studies, texture analysis with different imaging modalities has been shown to reflect tumor heterogeneity and was reported to be able to distinguish between multiple features—including imaging parameters, pathological subtypes, and genetic profiles—in various types of tumors (26–28). Therefore, in oncological imaging, textural analysis methods are already well established. Tumor heterogeneity can be caused by variations in cellularity, angiogenesis, extracellular matrix, or necrosis (29). Thus, quantification of ADC map histograms and definition of ADC threshold values for different diseases might represent a prospective, useful noninvasive imaging tool for differentiation of large inflammatory brain lesions. This should be further evaluated in future studies. Earlier differentiation could allow patients to be selected sooner and directed more quickly to the most appropriate treatment.

## Conclusion

ADC map histograms and ADC threshold values for different conditions may be useful for differentiation of large inflammatory brain lesions and further studies are merited.

## Author contributions

FW and AS collected all the references, wrote the main clinical part of the brief research report and contributed to the study design. FW, AC, and RW supervised the report. RH, JM, and NK were responsible for correction, and also contributed to the background clinical knowledge of our case. LG and AH contributed to the histogram analysis data collection. IM was in response for the histopathological statement. MH was in response for the laboratory findings and analysis.

### Conflict of interest statement

FW received a research grant from the Swiss MS Society, none related to the submitted work. RH received research and/or travel grants from Novartis, Biogen Idec and the Swiss MS Society and speaker's honoraria from Biogen, Novartis, Merk and Almirall, none related to this work. IM reports personal fees from BiogenIdec, Bayer Healthcare, TEVA, Serono, Novartis, Genzyme, and Roche as well as a grant from BiogenIdec, none related to the submitted work. AC received personal compensation for activities with Bayer, Biogen, Genzyme, Merck, Novartis, Roche, and Teva and research support from the Swiss National Science Foundation (SNF, No. 310030_172952), Genzyme and UCB. He serves on the editorial board of *Clinical and Translational Neuroscience* and the *Journal of International Medical Research*. AS received speaker's honoraria and/or travel compensation for activities with Almirall Hermal GmbH, Biogen, Merck, Novartis, Roche and Sanofi Genzyme, none related to this work. The remaining authors declare that the research was conducted in the absence of any commercial or financial relationships that could be construed as a potential conflict of interest.

## Abbreviations

ADC: apparent diffusion coefficient
CNS: central nervous system
CSF: cerebrospinal fluid
DWI: diffusion-weighted imaging
LETM: longitudinal extensive transverse myelitis
MS: multiple sclerosis
NMO: neuromyelitis optica
NMOSD: neuromyelitis optica spectrum disorder
OCB: oligoclonal bands
PCR: polymerase chain reaction
PLEX: plasma exchange
ROI: region of interest.
