# Supplementary material for: Rebound After Fingolimod and a Single Daclizumab Injection in a Patient Retrospectively Diagnosed With NMO Spectrum Disorder—MRI Apparent Diffusion Coefficient Maps in Differential Diagnosis of Demyelinating CNS Disorders
Source: Front Neurol. 2018 Sep 27;9:782. doi: 10.3389/fneur.2018.00782 (PMC6170610; doi:10.3389/fneur.2018.00782)
Supplement: Supplementary file 1 [file Data_Sheet_1.doc]

**Tables**

**TABLE 1.** ROI-based ADC values (10^-6^mm^2^/s) of the brain lesions in the NMOSD patient

|  | ROI mean ADC values lesion *10^−6^mm^2^/s] | Skewness | Kurtosis |
| --- | --- | --- | --- |
| No. of days after onset |  |  |  |
| 3 | 0.253 ± 0.002 | −0.760 | 2.947 |
| 7 | 0.526 ± 0.002 | 0.199 | 1.538 |
| 10 | 0.905 ± 0.003 | −0.273 | 3.077 |

Values are presented as mean ± SD. ROI includes all visually diffusion-restricted lesions.

**TABLE 2.** ROI-based ADC values (10^−6^mm^2^/s) of the brain lesions in the MS patient

|  | ROI mean ADC values  lesion *10^−6^mm^2^/s] | Skewness | Kurtosis |
| --- | --- | --- | --- |
| No. of days after onset |  |  |  |
| 1 | 0.211 ± 0.001 | −0.516 | 2.452 |
| 2 | 0.300 ± 0.001 | 0.442 | 2.108 |
| 3 | 0.554 ± 0.002 | 0.468 | 3.637 |
| 5 | 0.401 ± 0.001 | −0.198 | 3.464 |
| 9 | 0.462 ± 0.001 | −0.237 | 2.119 |

Values are presented as mean ± SD. ROI includes all visually diffusion-restricted lesions.

**TABLE 3.** ROI-based ADC values (10^−6^mm^2^/s) of infarction in the stroke patient.

|  | ROI mean ADC values  lesion *10^−6^mm^2^/s] | Skewness | Kurtosis |
| --- | --- | --- | --- |
| No. of days after onset |  |  |  |
| 1 | 0.390 ± 0.001 | 0.384 | 1.000 |
| 2 | 0.465 ± 0.001 | 0.432 | 1.321 |
| 5 | 0.390 ± 0.001 | 0.914 | 2.629 |

Values are presented as mean ± SD. ROI includes all visually diffusion-restricted lesions.
