# Supplementary material for: Rebound After Fingolimod and a Single Daclizumab Injection in a Patient Retrospectively Diagnosed With NMO Spectrum Disorder—MRI Apparent Diffusion Coefficient Maps in Differential Diagnosis of Demyelinating CNS Disorders
Source: Front Neurol. 2018 Sep 27;9:782. doi: 10.3389/fneur.2018.00782 (PMC6170610; doi:10.3389/fneur.2018.00782)
Supplement: Supplementary file 2 [file Data_Sheet_2.docx]

**Supplementary Figures captions**

**Sup. Figure S1**

**Brain biopsy with inflammatory demyelinating lesion and astrocytopathic changes.**

A cellular white matter lesion (HE, A) with confluent demyelination (LFB/PAS, showing an absence of blue myelin staining in B). The inflammatory infiltrate consists of numerous macrophages (CD163, D) and T cells (CD3, D). Only a few CD4-positive T helper cells are present (E). Strikingly, in some lesion areas astrocytes have been lost (GFAP, F). An aquaporin-4 loss is apparent (AQP4 G, higher magnification in H). For comparison, AQP4 expression in non-demyelinated areas is also shown (AQP4, I). Staining for the myelin protein CNP shows pale myelin with myelin degradation products present within macrophages, indicating early active demyelination (CNP, J + K; arrowheads show macrophages with CNP-positive degradation products). Apoptotic oligodendrocytes are numerous (K, arrows). Oligodendrocytes are absent within the lesion (L, p25, oligodendrocytes are absent in the lower part of the image within the demyelinating lesion). These histopathological changes are typical for neuromyelitis optica spectrum disorders.

Scale bar A–E, G, I–J, L: 500 µm; scale bar F, H, K: 100 µm.

**Sup Figure S2 to S5. MRI brain history for the NMOSD patient.**

The transverse FLAIR-attenuated inversion recovery weighted scans (top) and the transverse contrast-enhanced T1 weighted scans (lower row) for the NMOSD patient at different time points.

**Sup. Figure S6. MRI spine history for the NMOSD patient.**

The sagittal PD-fast spin-echo, the transverse T2 weighted scans and the transverse (resp. sagittal in the last MRI) contrast-enhanced T1 weighted scans demonstrating the non-enhancing PD and T2w hyperintense lesion at C3-C4.
