# Supplementary figures and images for: Rebound After Fingolimod and a Single Daclizumab Injection in a Patient Retrospectively Diagnosed With NMO Spectrum Disorder—MRI Apparent Diffusion Coefficient Maps in Differential Diagnosis of Demyelinating CNS Disorders
Source: Front Neurol. 2018 Sep 27;9:782. doi: 10.3389/fneur.2018.00782 (PMC6170610; doi:10.3389/fneur.2018.00782)

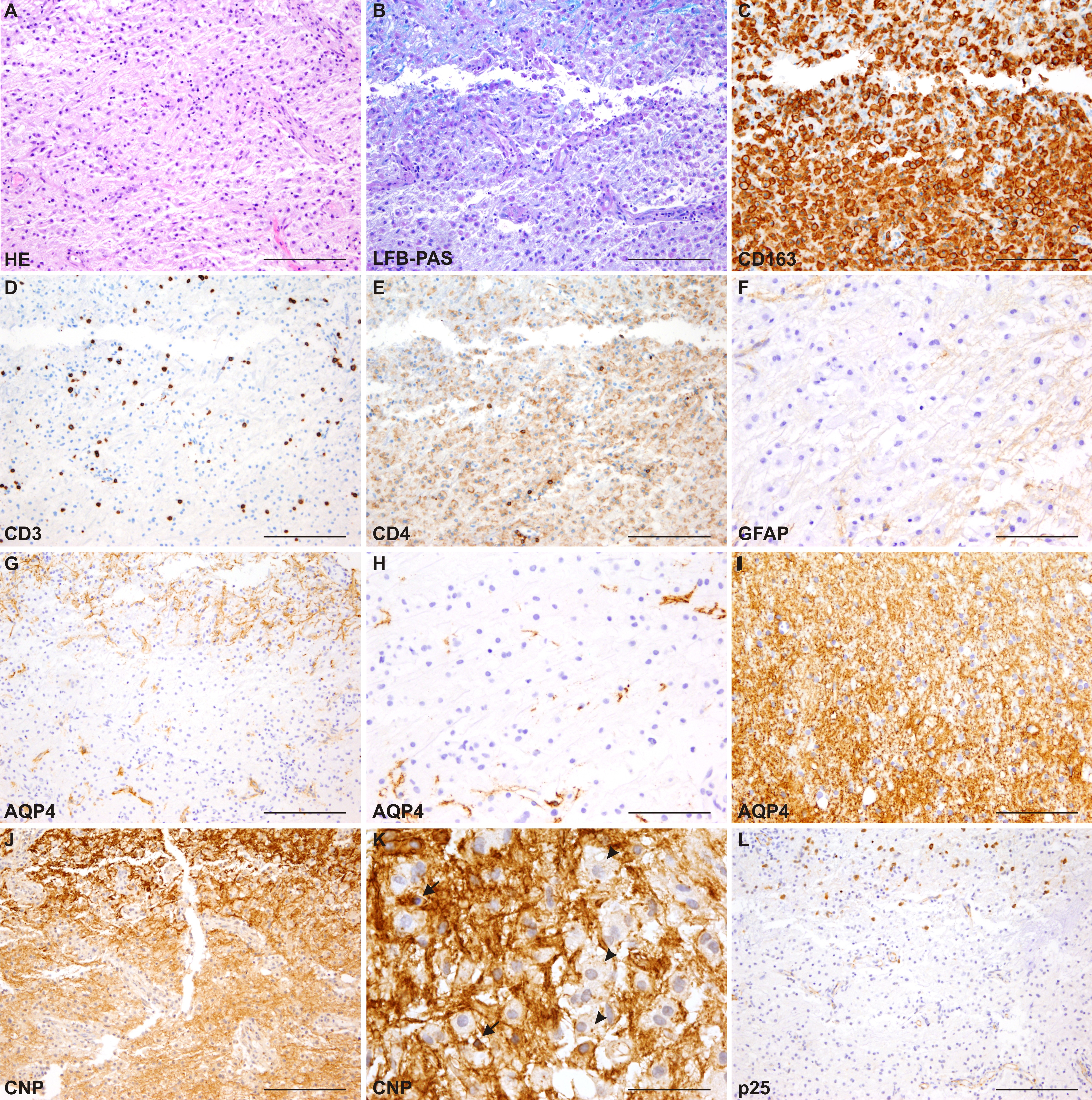

Supplement: Supplementary file 3 [file Image_1.JPEG]

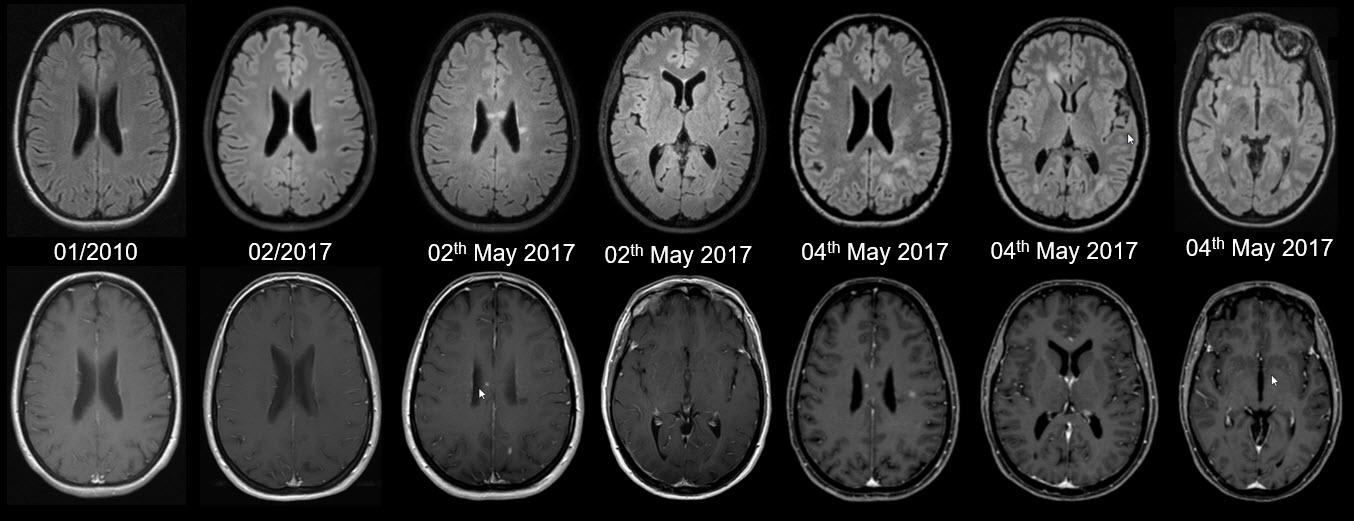

Supplement: Supplementary file 4 [file Image_2.JPEG]

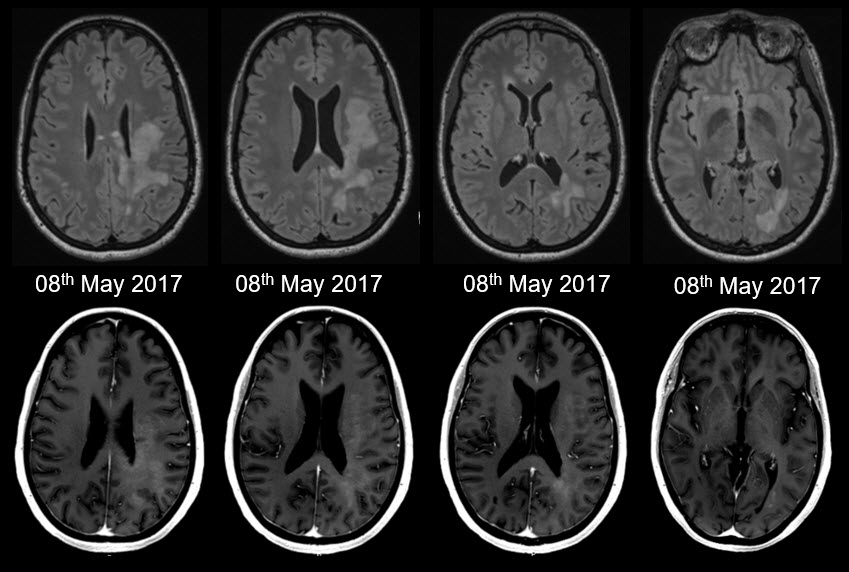

Supplement: Supplementary file 5 [file Image_3.JPEG]

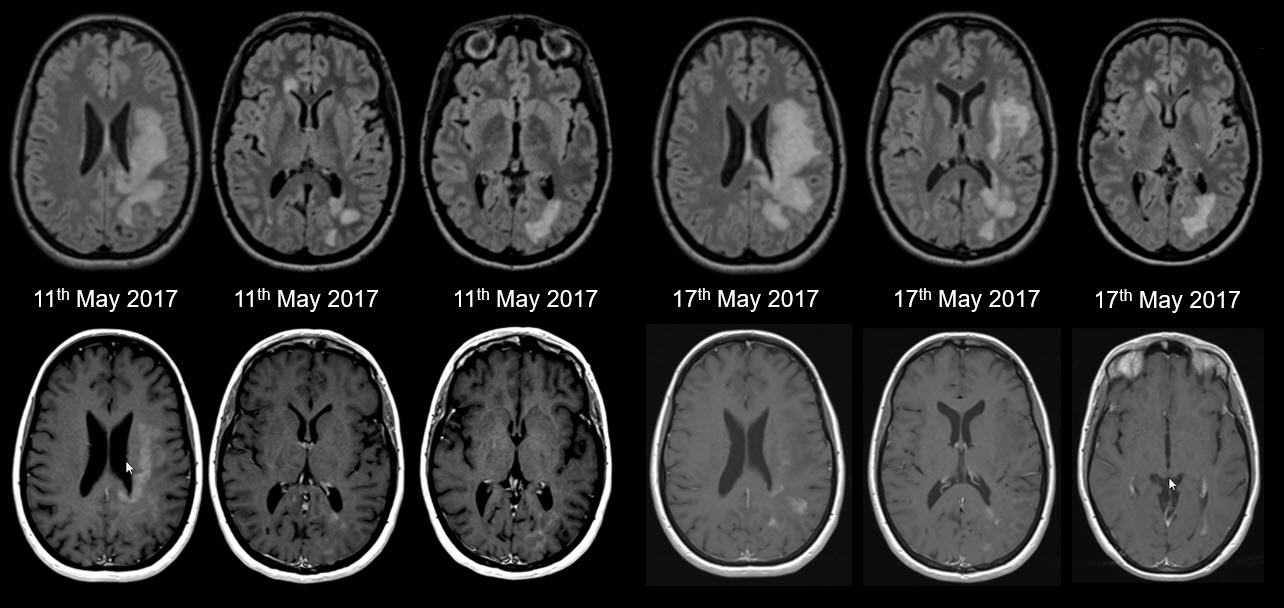

Supplement: Supplementary file 6 [file Image_4.JPEG]

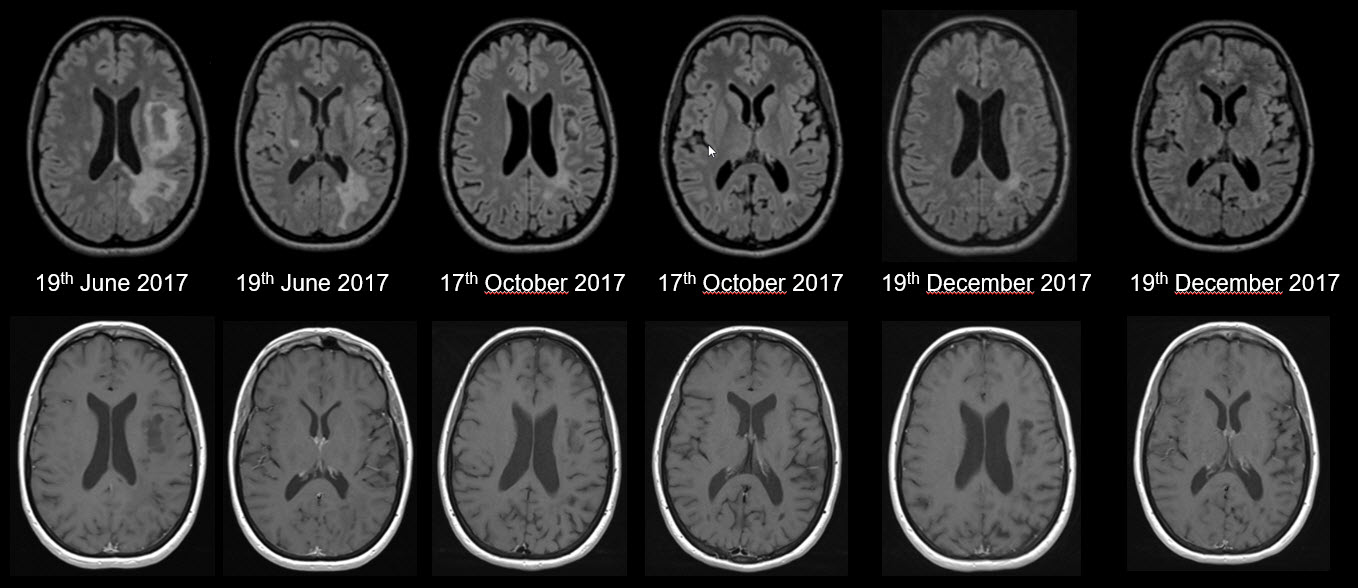

Supplement: Supplementary file 7 [file Image_5.JPEG]

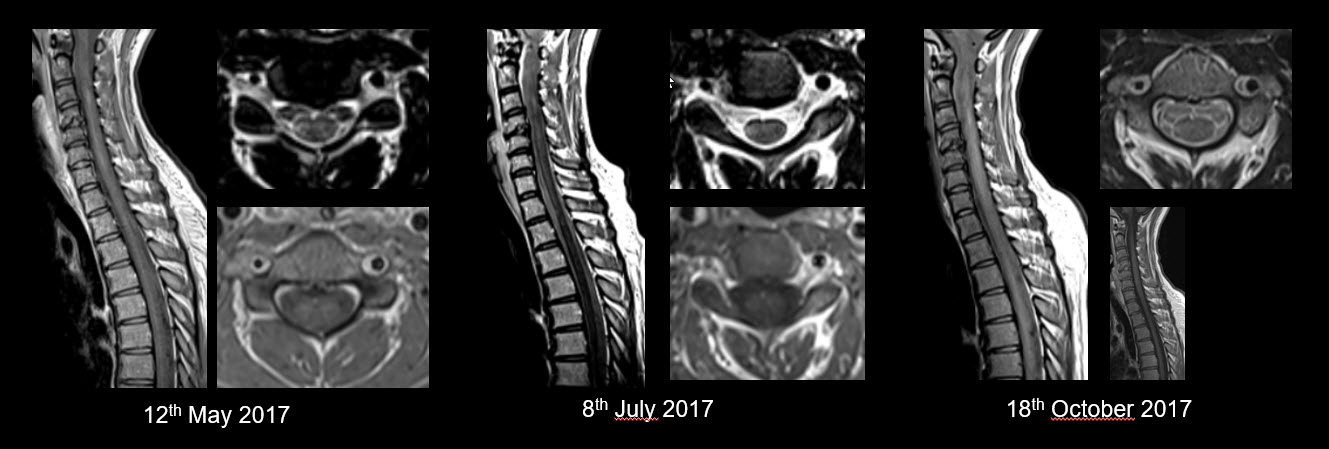

Supplement: Supplementary file 8 [file Image_6.JPEG]
